# Effect of Ethanol Stress on the Fatty Acid Ethyl Ester Biosynthesis Pathways of *Baijiu* Brewing Yeast

**DOI:** 10.3390/foods15071129

**Published:** 2026-03-25

**Authors:** Yanru Chen, Yin Wan, Wenqin Cai, Mengxiang Li, Guiming Fu

**Affiliations:** 1 State Key Laboratory of Food Science and Resources, Nanchang University, Nanchang 330047, China; chenyr0427@163.com (Y.C.); yinwan@ncu.edu.cn (Y.W.); caiwenqin@email.ncu.edu.cn (W.C.); l18438596263@126.com (M.L.); 2 School of Life Sciences, Qilu Normal University, Jinan 250200, China; 3 International Institute of Food Innovation Co., Ltd., Nanchang University, Nanchang 330299, China

**Keywords:** ethanol stress, ethyl acetate, ethyl hexanoate, gene expression, *Wickerhamomyces anomalus*

## Abstract

The flavor and scent of Chinese *Baijiu* are closely linked to the quantity of fatty acid ethyl esters, and their generation is closely associated with *Baijiu* brewing yeast, most notably ethyl acetate (EA) and ethyl hexanoate (EH). At present, however, the specific mechanism of EA and EH produced by *Wickerhamomyces anomalus* under ethanol stress during the brewing of *Baijiu* remains unclear. Our study findings revealed that ethanol stress inhibited the generation of precursor substances (pyruvate and acetyl-CoA) in the fatty acid ethyl ester biosynthesis pathway of *W. anomalus* NCU003. The high level of EA produced in the fatty acid ethyl ester biosynthesis pathway was associated with the enhanced expressions of *ATF1* and *EAT1* and the increased activity of C2 esterase under 3% and 6% ethanol stress. The lower EA content was related to the high expression of *IAH1* and low activity of C2 esterase under 9% ethanol stress. We also found that the expression of *ACC*, *FAS1*, *FAS2*, *EHT1*, and *EEB1* was up-regulated, which may promote the synthesis of EH under ethanol stress, whereas the activity of C6 esterase may have no effect on the synthesis of EH. Our study results indicated that the above genes and C2 esterase can be modulated in *W. anomalus* NCU003 under ethanol stress, thus promoting the synthesis of fatty acid ethyl esters during the brewing of *Baijiu*.

## 1. Introduction

As one of the six internationally renowned distilled spirits (alongside brandy, gin, rum, vodka, and whisky), Chinese *Baijiu* is popular among Chinese people [1]. Fatty acid ethyl esters are the main aroma component that determines the flavor of Chinese *Baijiu*, and ethyl acetate (EA) and ethyl hexanoate (EH) are considered to be two important fatty acid ethyl esters, providing a pleasant fruity aroma (apple and banana) that affects the organoleptic quality and flavor profile of *Baijiu* [2,3]. The effective production of EA and ethyl caproate during the process of *Baijiu* brewing is therefore an important prerequisite to ensure the regular operation of the Chinese *Baijiu* industry. Research has shown that fatty acid ethyl esters are primarily produced by *Baijiu* brewing yeast. In particular, the *Baijiu* brewing yeast *Wickerhamomyces anomalus* contributes significantly to the synthesis of EA and EH during the brewing process of Baijiu [4,5].

Fermentation strains, raw materials, and processes are all important factors that influence the composition of fatty acid ethyl esters during the *Baijiu* brewing process, and many studies have focused on modulating these factors to ensure the production of EA, EH, and other fatty acid ethyl esters [6,7]. Change in the *Baijiu* brewing environment is also an important factor that affects the production of fatty acid ethyl esters by *Baijiu* brewing yeast [8]. In addition, ethanol is the primary metabolite produced by yeast during the fermentation process. As it is continuously produced at increasing levels as fermentation progresses, high ethanol levels can exert stress on the yeast, thereby affecting its ability to produce aroma compounds [9]. The results of one study demonstrated that appropriate use of ethanol could promote the synthesis of EA and EH by *Saccharomyces cerevisiae*, thereby improving the overall flavor profile of wine [10]. In another study, ethanol stress also caused growth inhibition of *Kluyveromyces marxianus* but increased the content of fatty acid ethyl esters [11]. In addition, the results indicated that ethanol stress may significantly affect yeast-induced fatty acid ethyl ester biosynthesis during *Baijiu* brewing.

Pyruvate is an important substrate for the synthesis of fatty acid ethyl esters, primarily used for the synthesis of acetyl-CoA and hexanoyl-CoA [12]. In one study, researchers found a negative correlation between the changes in the content of pyruvic acid and EA, potentially due to the conversion of pyruvate into a greater number of precursor substances for the generation of EA in *S. cerevisiae* [13]. It is well established that fatty acid ethyl esters are primarily produced by the alcohol acetyltransferase (AATase) encoded by *ATF1*/*2*, *EEB1*, and *EHT1*, which can synthesize fatty acid ethyl esters by utilizing acetyl-CoA, hexanoyl-CoA, and ethanol. The ATF1/2 genes mainly regulate the synthesis of EA; in comparison, *EEB1* and *EHT1* are the regulatory genes involved in the synthesis of EH. Research has indicated that increased expression levels of *ATF1*, *ATF2*, *EHT1*, and *EEB1* can promote the generation of EA and EH in yeast [14]. Yeast can also produce fatty acid ethyl esters from ethanol and fatty acids through the role of esterase in the esterification process [13]. Thus, EA and EH content is primarily determined by the expression of genes related to fatty acid ethyl ester production and the activity of enzymes. Of note, in most current studies, researchers have focused exclusively on the impact of environmental stress on changes in aroma compound production by brewing yeast, without exploring the specific mechanisms by which environmental stress affects particular aroma compounds.

In our previous studies, the concentration of ethanol reached up to 9% (*v*/*v*) while brewing special-flavor *Baijiu*. Moreover, ethanol at different concentrations was found to inhibit the growth of special-flavor *Baijiu* brewing yeast (*W. anomalus* NCU003) [15,16]. Thus, the aim of this study was to investigate the effect of ethanol stress on the production of EA and EH by *W. anomalus* and to systematically elucidate the specific mechanisms by which ethanol stress affects the generation of EA and EH from the perspectives of genes, enzyme activities, and metabolites. It is hoped that these results can provide a theoretical basis for the directional regulation of fatty acid ethyl ester production by *Baijiu* brewing yeast in the future.

## 2. Materials and Methods

### 2.1. Construction of Ethanol Stress Fermentation Model

The *W. anomalus* NCU003 used in this study was sourced from Zhangshugong Wine and Spirits Co., Ltd. (Zhangshu, China) and exhibited high ester production capacity [17]. Pre-treatment of *W. anomalus* NCU003 was implemented based on the method employed in our previous work [18]. We continued to cultivate activated *W. anomalus* NCU003 (1% vaccination rate) by using malt extract medium (0.13 kg malt extract and 100 mg chloramphenicol were dissolved in 1000 mL distilled water) for 8 h. Thereafter, additional anhydrous ethanol was added to the culture medium of *W. anomalus* NCU003 to achieve ethanol concentrations of 3% (*v*/*v*), 6% (*v*/*v*), and 9% (*v*/*v*). No treatment was administered to the control group. Lastly, cultivation was continued in the shaker (28 °C and 160 rpm) for 14 h, *W. anomalus* NCU003 cells were removed, and the indexes were measured. During this period, samples were taken for the determination of various indicators.

### 2.2. Determination of Residual Sugar and Ethanol Contents

The residual sugar content was determined based on the method of Piao et al. [19], and ethanol content was determined based on the method of He et al. [20]. The content of residual sugar and ethanol was expressed as g/L and %, respectively.

### 2.3. Analysis of EA and EH Contents via GC-FID

The analysis of EA and EH contents was performed using a gas chromatograph (Agilent Technologies, Santa Clara, CA, USA) equipped with an HP-5 column (30 m × 0.25 mm × 0.25 µm). The fermentation broth of *W. anomalus* NCU003 (1 mL) was centrifuged (4 °C, 12,000× *g*, 10 min), and the supernatant was collected and filtered through a 0.22 µm microporous membrane prior to analysis.

The analysis procedure involved the following parameters: detector, FID; carrier gas, nitrogen; carrier gas flow rate, 1 mL/min; injector temperature, 230 °C; detector temperature, 250 °C; injection volume, 1 µL; split ratio, 15:1. The initial temperature was set at 35 °C and held for 5 min, then increased to 96 °C at a rate of 8 °C/min and maintained for 3 min, followed by an increase to 110 °C at 2 °C/min with no hold, and finally increased to 240 °C at 15 °C/min and held for 3 min. The contents of EA and EH were determined with reference to standard substances (Aladdin Biochemical Technology Co., Ltd., Shanghai, China) and were expressed as mg/L.

### 2.4. Analysis of Acetic Acid and Caproic Acid Contents

First, 100 mg of the *W. anomalus* NCU003 cells was extracted with 100 μL ultrapure water, and 5 mol/L HCl was used to adjust the pH to 2.5. Thereafter, the mixtures were centrifuged (4 °C, 12,000× *g*, 15 min), with only the supernatant retained for use in the analysis of acetic acid and caproic acid content.

The supernatant was filtered through a 0.22 μm microporous membrane. The analysis procedure for acetic acid and caproic acid involved the use of a gas chromatograph (Agilent Technologies, USA) equipped with a DB-FAT WAXUI column (30 m × 0.25 mm × 0.25 µm), with the procedure involving the following: detector, FID; carrier gas, nitrogen; carrier gas flow rate, 3 mL/min; injector temperature, 220 °C; detector temperature, 250 °C; injection volume, 1 µL; split ratio, 10:1. The initial temperature was set at 60 °C and held for 5 min, then increased to 160 °C at a rate of 10 °C/min and maintained for 2 min, followed by an increase to 220 °C at 20 °C/min and held for 3 min. The contents of acetic acid and caproic acid were determined with reference to standard substances (Aladdin Biochemical Technology Co., Ltd., China) and were expressed as μg/g.

### 2.5. Analysis of Pyruvate and Acetyl-CoA Contents

The determination of acetyl-CoA content was performed based on the instructions of the acetyl-CoA content assay kit (Beijing Solarbio Science and Technology Co., Ltd., Beijing, China). First, 100 mg of the *W. anomalus* NCU003 cells was added to 0.3 mL of phosphate-buffered solution (pH 7.2) and ultrasonically crushed on ice for 150 s. The supernatant was obtained via centrifugation (4 °C, 12,000× *g*, 10 min) and used for the determination of pyruvate content, with reference to the method of Metrani et al. [21]. The contents of acetyl-CoA and pyruvate were expressed as μmol/g and μg/g, respectively.

### 2.6. Determination of Related Enzyme Activities

The determination of pyruvate decarboxylase (PDC) activity was performed based on the instructions of the PDC activity assay kit (Beijing Solarbio Science and Technology Co., Ltd., China), and the determination of acetyl-CoA synthetase (ACS) activity was performed based on the method of Zheng et al. [22]. The reaction mixture for ACS contained 100 mmol/L Tris-HCl buffer (pH 8.0), 0.005 mol/L NAD+, 0.2 mmol/L CoA, 0.01 mol/L malic acid, 0.01 mol/L ATP, 100 U/mL malate dehydrogenase, 3 U/mL citrate synthase, and 2 mL crude enzymes, and absorbance was measured at 340 nm. The activities of PDC and ACS were expressed as U/g. The determination of C2 esterase and C6 esterase activities was performed based on the method of Matthews et al. [23]. The reaction mixture for C2 and C6 contained 25 mmol/L p-Nitrophenyl acetate (for the determination of C2 activity), 25 mmol/L p-Nitrophenyl hexanoate (for the determination of C6 activity), citric acid–phosphate buffer (pH 5.0), and 0.5 mol/L NaOH. The activities of C2 and C6 were expressed as U/g.

### 2.7. Quantitative PCR Analysis

The extraction of total RNA from *W. anomalus* NCU003 cells, the synthesis of cDNA, and qPCR analysis were performed based on the method of Chen et al. [18]. The primer sequences of key genes involved in the biosynthesis pathway of fatty acid ethyl esters are presented in Table S1. The *ACT1* gene was used as a control gene, and the 2^−ΔΔCT^ method was used to calculate the relative expression levels of each gene.

### 2.8. Data Analysis

All experiments in this study were subjected to three biological replicates. The software Origin 2021 and SPSS 21.0 were used to produce figures and conduct statistical analyses. Significant difference analysis (*p* < 0.05) was conducted using single-factor analysis and Duncan’s test.

## 3. Results

### 3.1. The Changes in Residual Sugar and Ethanol Contents in the Fermentation Broth of W. anomalus NCU003

**Figure 1:**
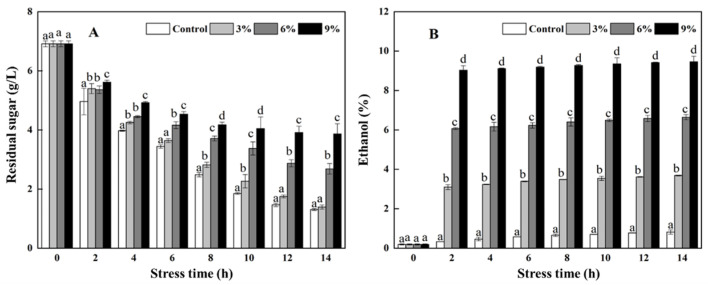
Effects of ethanol stress on the residual sugar (**A**) and ethanol (**B**) contents in the fermentation broth. Note: different letters represent a significant difference (*p* < 0.05) in the content at the same stress time.

As shown in Figure 1A, the content of residual sugar in the fermentation broth decreased as the stress time increased. Moreover, the rate of decrease in the content of residual sugars slowed with the increase in ethanol concentration. The residual sugar content remained significantly (*p* < 0.05) higher in the 9% ethanol stress group than in the other groups. The ethanol content in all groups increased (*p >* 0.05) slightly with the increase in stress time, and newly generated ethanol content decreased with the increase in ethanol stress concentrations (Figure 1B). The ethanol content in the control, 3% ethanol stress, 6% ethanol stress, and 9% ethanol stress groups was 0.82%, 3.69%, 6.65%, and 9.46% at 14 h after ethanol stress, respectively.

### 3.2. The Changes in EA and EH Contents of W. anomalus NCU003

**Figure 2:**
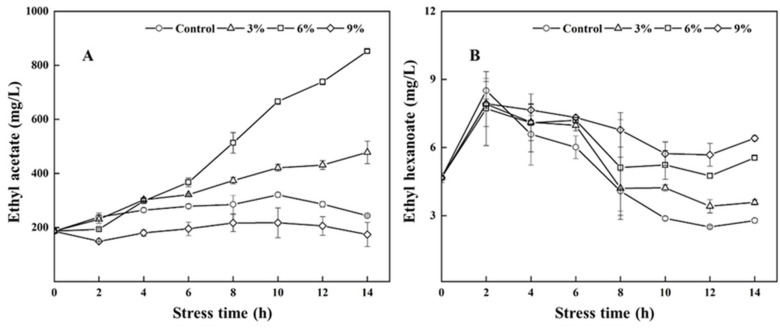
Effects of ethanol stress on the ethyl acetate (**A**) and ethyl hexanoate (**B**) contents of *W. anomalus* NCU003.

The content of EA in the 3% ethanol stress and 6% ethanol stress groups was higher than that of the group without ethanol supplementation, and the highest EA content was recorded in the 6% ethanol stress group at 14 h, a 3.50-fold increase compared to that of the control. Of note, the content of EA in the 9% ethanol stress group was lower than that of the group without ethanol addition during the entire fermentation period (Figure 2A). Moreover, ethanol supplementation promoted the production of EH by *W. anomalus* NCU003 in a concentration-dependent manner (Figure 2B).

### 3.3. The Changes in Gene Expression in the EA Biosynthesis Pathway of W. anomalus NCU003

**Figure 3:**
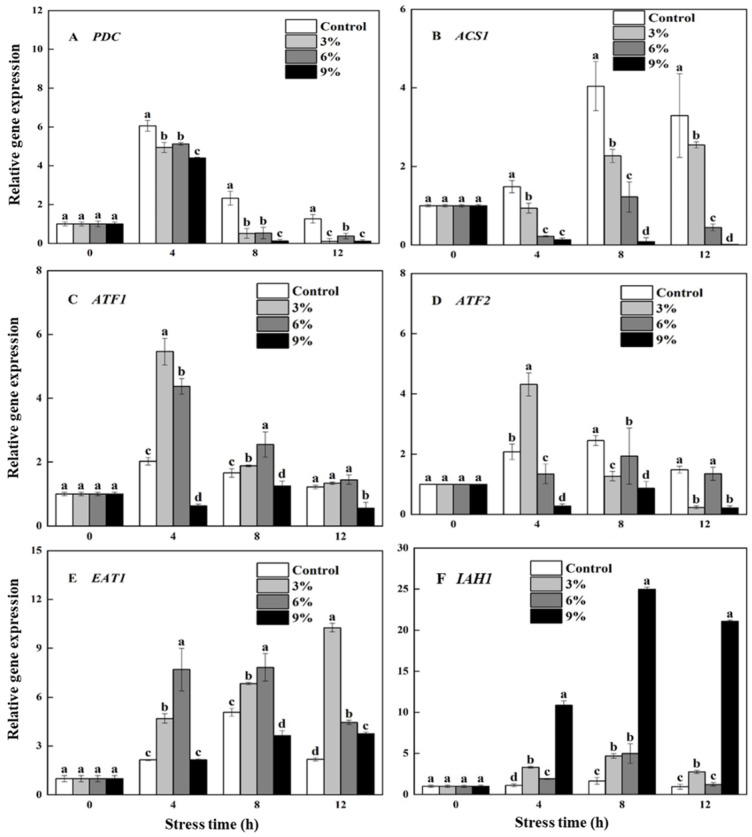
Effects of ethanol stress on the expression of *PDC* (**A**), *ACS1* (**B**), *ATF1* (**C**), *ATF2* (**D**), *EAT1* (**E**), and *IHA1* (**F**) in *W. anomalus* NCU003. Note: Different letters represent a significant difference (*p* < 0.05) within the same time period.

As shown in Figure 3A,B, ethanol supplementation significantly (*p* < 0.05) inhibited the expression level of *PDC* and *ACS1*. The expression levels of the *PDC* and *ACS1* genes in the 9% ethanol stress group amounted to only 5.5% and 2.1% of those in the control group at 8 h. The expression of *ATF1* and *EAT1* increased during the fermentation process under 3% and 6% ethanol stress (Figure 3C,E), and the expression of *ATF2* was only up-regulated in the early stage of fermentation under 3% ethanol stress (Figure 3D). However, the expression of *ATF1* and *ATF2* was significantly (*p* < 0.05) down-regulated during the entire fermentation process under 9% ethanol stress. The expression of *IAH1* was up-regulated after ethanol stress, and its expression was significantly (*p* < 0.05) up-regulated under 9% ethanol stress, a 21.84-fold increase compared to that of the control at 12 h (Figure 3F).

### 3.4. The Changes in the PDC and ACS Activities of W. anomalus NCU003

**Figure 4:**
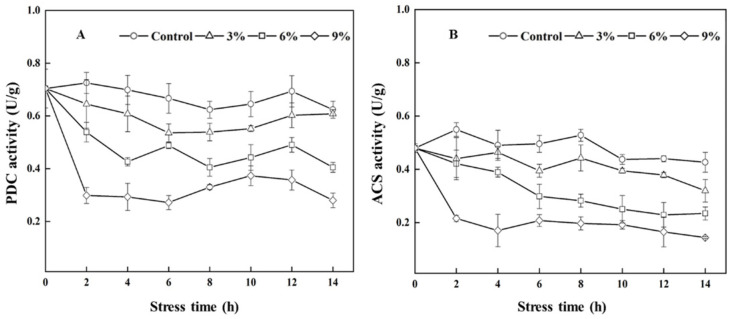
Effects of ethanol stress on the PDC (**A**) and ACS (**B**) activities of *W. anomalus* NCU003.

As shown in Figure 4, the changes in PDC and ACS activities ultimately remained consistent with the changes in *PDC* and *ACS1* gene expression levels (Figure 3A,B). The presence of ethanol reduced the activities of PDC and ACS in a concentration-dependent manner.

### 3.5. The Changes in Gene Expression in the EH Biosynthesis Pathway of W. anomalus NCU003

**Figure 5:**
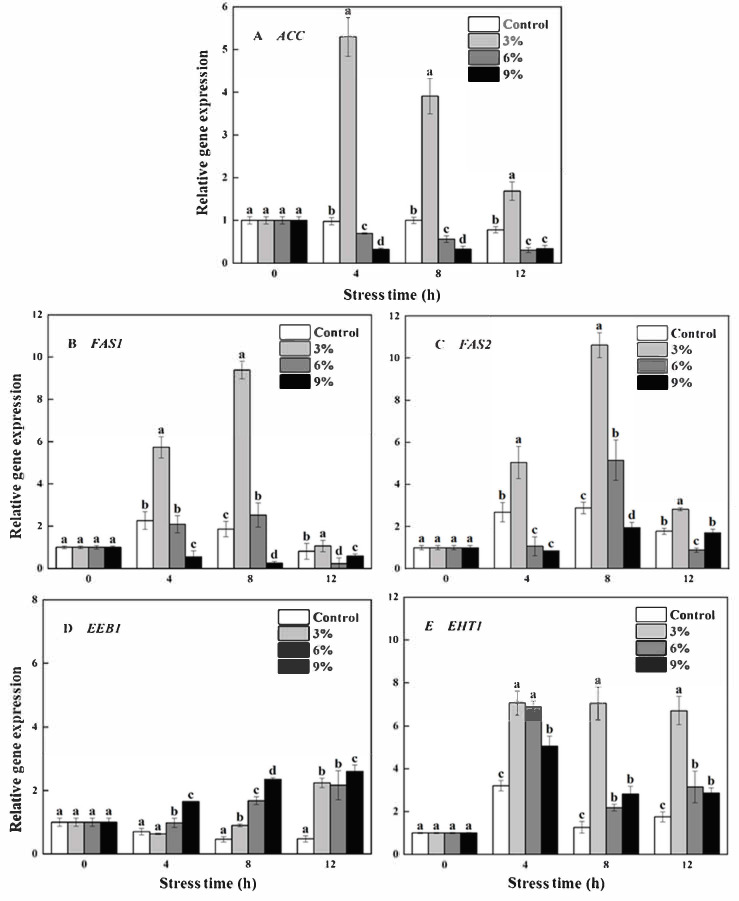
Effects of ethanol stress on the expression of *ACC* (**A**), *FAS1* (**B**), *FAS2* (**C**), *EEB1* (**D**), and *EHT1* (**E**) genes in *W. anomalus* NCU003. Note: Different letters represent a significant difference (*p* < 0.05) within the same time period.

The expression of *ACC* (Figure 5A), *FAS1* (Figure 5B), and *FAS2* (Figure 5C) was significantly (*p* < 0.05) up-regulated and down-regulated under 3% and 9% ethanol stress, respectively. As shown in Figure 5D,E, the expressions of *EEB1* and *EHT1* were significantly (*p* < 0.05) up-regulated by ethanol stress during the entire fermentation process. The expression of *EEB1* was concentration-dependent, with the greatest expression of *EEB1* recorded under 9% ethanol stress at 12 h, a 5.51-fold increase compared to that of the control. However, the greatest expression of *EHT1* was recorded under 3% ethanol stress at 8 h, a 5.57-fold increase compared to that of the control.

### 3.6. The Changes in the C2 Esterase and C6 Esterase Activities of W. anomalus NCU003

**Figure 6:**
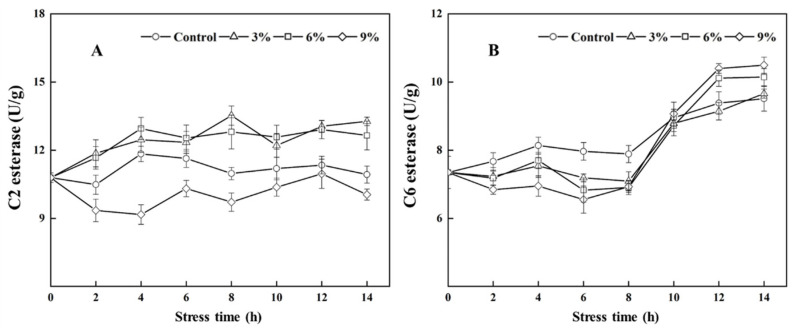
Effects of ethanol stress on the C2 esterase (**A**) and C6 esterase (**B**) activities of *W. anomalus* NCU003.

As shown in Figure 6A, compared with the group with no ethanol supplementation, the activity of C2 esterase decreased under 9% ethanol stress; in comparison, the activity of C2 esterase increased under 3% and 6% ethanol stress. Among the groups, the activity of C2 esterase in the 3% ethanol stress group was 1.23 times higher than that of the control group at 8 h post-treatment. Ethanol stress inhibited the activity of C6 esterase during the first 0–8 h. The activity of C6 esterase in all of the groups exhibited an upward trend during the remaining stress period; of note, the fastest increase in C6 esterase activity was observed under 9% ethanol stress, and the activity of C6 esterase in the 9% ethanol stress group was 1.10 times greater than that of the control group at 14 h after ethanol stress (Figure 6B).

### 3.7. The Changes in the Main Metabolite Content of W. anomalus NCU003

**Figure 7:**
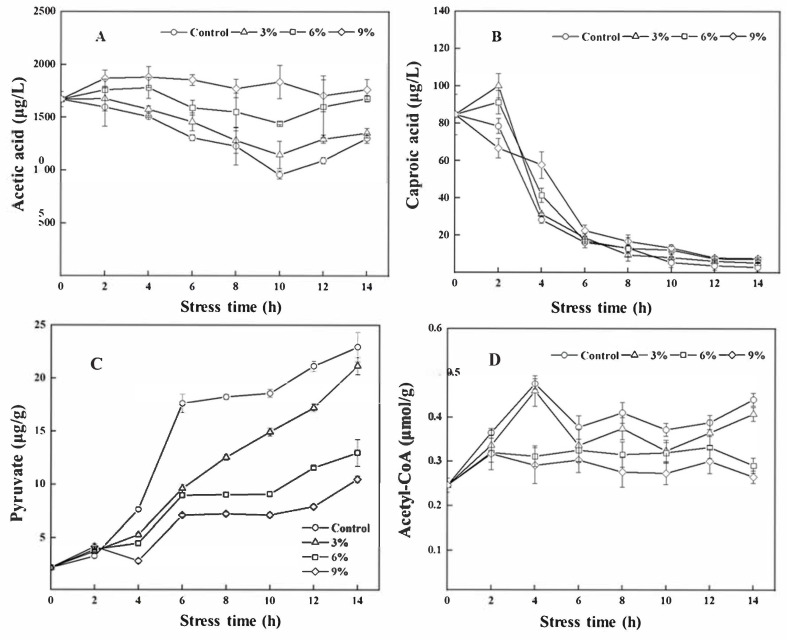
Effects of ethanol stress on the content of acetic acid (**A**), caproic acid (**B**), pyruvate (**C**), and acetyl-CoA (**D**) of *W. anomalus* NCU003.

As shown in Figure 7A, ethanol supplementation was found to promote the generation of acetic acid during the entire fermentation process, with the effect being concentration-dependent. Among the stress groups, the content of acetic acid in the 9% ethanol stress group was 1.92 times higher than that of the control group at 10 h after ethanol stress. The content of caproic acid exhibited a downward trend after 2 h; the content of caproic acid remained consistently high during the remaining fermentation process under 9% ethanol stress, and the content of caproic acid in the 9% ethanol stress group was 2.04 times higher than that of the control group at 4 h after ethanol stress. (Figure 7B). Moreover, ethanol stress inhibited the generation of pyruvate and acetyl-CoA content in *W. anomalus* NCU003 and was concentration-dependent (Figure 7C,D). Among the groups, the content of pyruvate in the control group was 2.19 times higher than that of the 9% ethanol stress group at 14 h after ethanol stress.

## 4. Discussion

EA and EH are produced through microbial metabolism (primarily by ester-producing yeast) during the *Baijiu* brewing process, and they play a dominant role in influencing its sensory quality. Notably, *W. anomalus* serves as the main ester-producing yeast [24]. The results of a number of studies have also demonstrated that different environmental stress factors can enhance the ability of yeast to produce EA and EH [10,11]. For example, the growth of *Pichia kudriavzevii* was inhibited under organic acid stress but ultimately increased the EA content during the brewing process of sea buckthorn wine [25]. From these results, it can therefore be concluded that ethanol stress may result in the generation of a greater number of aroma components during the brewing process of *Baijiu*. An in-depth study of EA and EH production mechanisms under ethanol stress would be advantageous in advancing *W. anomalus* NCU003’s potential application in the *Baijiu* brewing process.

Yeast can use glucose to produce pyruvate and acetic acid via the glycolytic pathway during the brewing process, which are key precursors for acetyl-CoA synthesis [26]. Our results indicated that the utilization rate of residual sugar in fermentation broth gradually decreased, and the production of pyruvate was gradually inhibited with the increase in ethanol concentration. This phenomenon indicated that ethanol stress may reduce the glucose uptake of *W. anomalus* NCU003, decrease the carbon metabolism of the glycolysis pathway, and inhibit the synthesis of pyruvate.

The pyruvate decarboxylase (encoded by PDC) is responsible for converting pyruvate into acetic acid; then, acetic acid is converted into acetyl-CoA under the action of acetyl-CoA synthetase (encoded by ACS1) [27]. Both *PDC* and *ACS1* genes are key genes for synthesizing EA. The *ACS1* gene plays a particularly important role, with the results of one study indicating that the expression of *ACS1* was positively correlated with EA content. For example, the expression of *ACS1* in *S. cerevisiae* was inhibited under hypertonic stress, resulting in a reduction in EA synthesis [28]. Our results indicate that the expression of *PDC* and *ACS1* genes and the activity of PDC and ACS were significantly inhibited following ethanol supplementation, resulting in a reduction in acetyl-CoA content. Of note, we found that the acetic acid content of *W. anomalus* NCU003 increased in all ethanol stress groups, which may be related to ethanol stress inhibiting ACS activity, thereby preventing acetic acid from being fully utilized to synthesize acetyl-CoA and thus resulting in the accumulation of acetic acid under ethanol stress.

Nevertheless, our results indicated that the content of EA was higher than that of the group without ethanol supplementation under 3% and 6% ethanol stress, which may be the result of an increase in *ATF1*/*2* gene expression. AATase (ATF1/2 encoding) can catalyze the combination of ethanol and acetyl-CoA, thus completing the most critical step in the production of EA [29]. Our results showed that overexpression of *ATF1* and *ATF2* could enhance the environmental tolerance of *Saccharomyces pastorianus* and promote the synthesis of EA [14] and also showed that the effect of *ATF2* may be less pronounced than that of *ATF1* [30]. The expression of *ATF2* was higher only in the early stage of 3% ethanol stress. Our results indicated that *ATF1* showed stronger induction than *ATF2* in the production of EA under 3% and 6% ethanol stress. Similarly, the *EAT1* gene is a recently discovered coding gene of AATase in yeast. In one study, the authors found that *Kluyveromyces marcianus’s* ability to produce EA significantly decreased after the deletion of the *EAT1* gene, thus confirming that the *EAT1* gene is also a key gene for synthesizing EA [31]. The expression of the *EAT1* gene was found to be up-regulated under 3% and 6% ethanol stress. Overall, our results showed that *W. anomalus* NCU003 could promote the production of EA by up-regulating the expression of *ATF1* and *EAT1* under 3% and 6% ethanol stress.

In addition, the isoamyl acetate-hydrolyzing esterase (*IAH1* encoding) can degrade EA and reduce the EA content in yeast [32]. In one study, the authors found that the ability to produce EA was significantly reduced after overexpression of the *IAH1* gene in *S. cerevisiae*, which confirmed the role of this gene in reducing the EA content [33]. Our results showed that the expression of *IAH1* also exhibited an upward trend with increased ethanol content. From these results, it can therefore be concluded that *W. anomalus* NCU003 could promote the production of EA by up-regulating *EAT1* expression under 9% ethanol stress; however, the *IAH1* gene may play a more important role in the degradation of EA, resulting in the lowest EA content in the 9% ethanol stress group.

Of note, the authors of some studies have found a close relationship between esterase activity and the synthesis and accumulation of EA and EH in yeast [34,35]. Sumby et al. [36] investigated the activity of C2~C10 esterases in *Oenococcus oeni* and found that they exhibited specificity for the reaction of fatty acid substrates with different carbon chain lengths, which determined the composition of fatty acid ethyl esters, and their content was inversely proportional to the length of the carbon chain. Among them, C2 and C6 esterases can directly esterify fatty acids and ethanol to produce EA and EH, respectively [36]. In one study, it was found that the enhancement of C2 and C6 esterase activity after ARTP mutation aided *S. cerevisiae* in producing greater amounts of EA and EH and improved the flavor profile of jackfruit wine [37]. In this study, our results indicated that 3% and 6% ethanol stress increased C2 esterase activity in *W. anomalus* NCU003; however, 9% ethanol inhibited C2 esterase activity. These results were consistent with the change rule of EA. Moreover, acetic acid serves as a key substrate for the production of EA catalyzed by C2 esterase. The result also showed that the acetic acid content was relatively high compared with other groups under 9% ethanol stress, suggesting that the decrease in C2 esterase activity could prevent *W. anomalus* NCU003 from fully utilizing acetic acid to synthesize EA.

Hexanoyl-CoA and ethanol are also important substrates in the synthesis of EH. Acetyl-CoA carboxylase (ACC encoding) and fatty acid synthase (*FAS1* and *FAS2* encoding) play key roles in the synthesis of hexanoyl-CoA. ACC can convert acetyl-CoA to malonyl-CoA, and FAS can continue to convert acetyl-CoA and malonyl-CoA to hexanoyl-CoA, thus providing precursors for the production of EH [38]. The authors of one study found that the production of EH in Nong-flavor *Baijiu* could be significantly increased by overexpressing *ACC*, *FAS1*, and *FAS2* genes in *S. cerevisiae*, respectively [39]. Their findings also indicated that the expression of these genes was significantly up-regulated under 3% ethanol stress, with this factor potentially explaining the high content of EH.

*EHT1* and *EEB1* genes can also encode ATT in yeast, which is the key enzyme in the production of medium-chain fatty acid ethyl esters. The results of one study indicate that EH content was significantly decreased in wine after either the *EHT1* or *EEB1* gene in *S. cerevisiae* was knocked out [40]. In addition, the results of another study also showed that *EHT1* and *EEB1* genes had positive effects on the synthesis of EH following the construction of recombinant strains by overexpressing *EHT1* and *EEB1* genes in *Pichia pastoris* [41]. The results showed that ethanol could increase the expression of *EHT1* and *EEB1*, with 3% ethanol mainly up-regulating the expression of *EHT1* and 6% and 9% ethanol mainly up-regulating the expression of *EEB1*. These findings suggest that the activity of AATase in *W. anomalus* NCU003 may be regulated by different genes under ethanol stress at different concentrations, with the regulatory capacity of the *EEB1* gene being greater than that of the *EHT1* gene. Therefore, the content of EH was relatively high under 6% ethanol stress and 9% ethanol.

Caproic acid is also a key substrate for the production of EH. We found that the contents of caproic acid and EH showed an overall decreasing trend, and the results also showed that the activity of C6 esterase increased under 9% ethanol stress at the middle and late stages of fermentation; however, the utilization of caproic acid was lower than that of other groups. Our results suggested that there was no obvious regularity in the changes in caproic acid, C6 esterase, and EH under ethanol stress. Other research groups have found that microorganisms mainly affect the synthesis of ester compounds by acting on esterase with short carbon chains during the brewing process. Moreover, the difference in metabolic ability among different strains will also impact the activity of esterase and the specificity of substrates, thus affecting the content of EA and EH [34,37]. Such findings therefore indicate that the contribution of C6 esterase to the production of EH may be far less significant than that of *EEB1* and *EHT1* genes in *W. anomalus* NCU003 and may not generate EH through the esterification reaction between caproic acid and ethanol under ethanol stress.

## 5. Conclusions

Our study revealed the specific biosynthesis mechanism of EA and EH produced by *Baijiu* brewing yeast under ethanol stress. Our results suggest that ethanol at different concentrations inhibited the expression of *PDC* and *ACS1*, with the activity of PDC and ACS related to the synthesis of acetyl-CoA; however, it increased the content of EA under 3% and 6% ethanol stress by enhancing the expression of *ATF1* and *EAT1* and the activity of C2 esterase. In addition, high expression of *IAH1* related to the catabolism of EA resulted in lower EA content under 9% ethanol stress. Our results also suggest that the production of EH was associated with the high expression of *ACC*, *FAS1*, *FAS2*, and *EHT1* under 3% ethanol stress. Moreover, the production of EH was associated with the high expression of *EEB1* under 6% and 9% ethanol stress. To conclude, moderate ethanol concentrations (3% and 6%, *v*/*v*) could promote the generation of EA and EH in *W. anomalus*. Therefore, modulating ethanol concentration during controlled *Baijiu* fermentation could represent an effective strategy for optimizing its flavor profile.

## Data Availability

The original contributions presented in this study are included in the article/Supplementary Materials. Further inquiries can be directed to the corresponding author.

## Supplementary Materials

The following supporting information can be downloaded at https://www.mdpi.com/article/10.3390/foods15071129/s1, Table S1: List of primers used in real-time PCR analysis.

## Abbreviations

The following abbreviations are used in this manuscript:

EA	Ethyl Acetate
EH	Ethyl Hexanoate
ARTP	Atmospheric and Room Temperature Plasma
GC-FID	Gas Chromatograph–Flame Ionization Detector
