# Supplementary material for: Effect of Ethanol Stress on the Fatty Acid Ethyl Ester Biosynthesis Pathways of Baijiu Brewing Yeast
Source: Foods. 2026 Mar 25;15(7):1129. doi: 10.3390/foods15071129 (PMC13073148; doi:10.3390/foods15071129)
Supplement: Supplementary file 1 [file foods-15-01129-s001.zip › foods-4168212-supplementary.pdf]

Effect of ethanol stress on fatty acid ethyl ester biosynthesis pathways of *Baijiu* brewing yeast

Yanru Chen[1,2], Yin Wan[1], Wenqin Cai[1], Mengxiang Li[1], Guiming Fu[1,*]

[1] State Key Laboratory of Food Science and Resources & College of Food Science and Technology, Nanchang University, Nanchang 330047, China

[2] School of Life Sciences, Qilu Normal University, Jinan, 250200, China.

* *Corresponding author:* State Key Laboratory of Food Science and Resources & College of Food Science and Technology, Nanchang University, Nanchang 330047, China

E-mail address: fuguiming@ncu.edu.cn (GM. Fu).

Table S1 List of primers used in real-time PCR analysis.

Genes	Primer sequence Forward: 5´-3´	Primer sequence Reverse: 5´-3´
ACS1	CTAGATGGCCAAACGAGCCA	AGACAACAGTAGTGGCACCG
ACC	TCGTGGTCCTGCTCCAGATA	AGCAGCAACACGTAGTCTCC
ATF1	GTCGCTGTGGAGAAAGGTCA	TGGAGCACTTTCAGGGACAC
ATF2	CTAGATGGCCAAACGAGCCA	AGACAACAGTAGTGGCACCG
IAH1	TGGTCCACAAATTGTTCCCCT	AGCTTCTCTTGATGGAGCCC
PDC	AACTATGCTGTGTGGGTGGG	AGCAGAGTGACGAGAAGCAC
EHT1	GACGAGAAGGCGACACATC	CCACTTGAAATCTCCCACTG
EEB1	GACTGGCCTAGACAACACCC	TTCACTGAACTTGCGGCTCT
EAT1	AAACGTTTCAGCATGGCCAAC	AACTTGTTCAGCGGTTGGTG
FAS1	TCTCCAGCTGCTAAACAGGC	TTGAGCAGCAACTGGACCAT
FAS2	TACACCCCTGATCCTGCTGA	TCTACCTGCACCTAGACCCC
ACT1	CGTCTGGATTGGTGGTTCTA	GTGGTGAACGATAGATGGAC
